# Cost-Effectiveness Study of Double-Flange Voice Prostheses in the Treatment of Periprosthetic Leakage in Laryngectomized Patients

**DOI:** 10.3390/jpm13071064

**Published:** 2023-06-29

**Authors:** Pedro Rodríguez-Lorenzana, Miguel Mayo-Yáñez, Carlos M. Chiesa-Estomba, Luigi Angelo Vaira, Jérôme R. Lechien, Antonino Maniaci, Irma Cabo-Varela

**Affiliations:** 1 Otorhinolaryngology—Head and Neck Surgery Department, Complexo Hospitalario Universitario A Coruña (CHUAC), 15006 A Coruña, Spain; plorenro@gmail.com (P.R.-L.); irma.cabo.varela@sergas.es (I.C.-V.); 2 Young-Otolaryngologists of the International Federations of Oto-Rhino-Laryngological Societies (YO-IFOS) Study Group, 75000 Paris, France; chiesaestomba86@gmail.com (C.M.C.-E.); luigi.vaira@gmail.com (L.A.V.); jerome.lechien@umons.ac.be (J.R.L.);; 3 Otorhinolaryngology—Head and Neck Surgery Department, Hospital Universitario Donostia—Biodonostia Research Institute, 20014 Donostia, Spain; 4 Maxillofacial Surgery Operative Unit, Department of Medical, Surgical and Experimental Sciences, University of Sassari, 07100 Sassari, Italy; 5 Department of Otolaryngology, Polyclinique de Poitiers, Elsan Hospital, 86000 Poitiers, France; 6 Department of Otolaryngology—Head & Neck Surgery, Foch Hospital, School of Medicine, UFR Simone Veil, Université Versailles Saint-Quentin-en-Yvelines (Paris Saclay University), 91190 Paris, France; 7 Department of Human Anatomy and Experimental Oncology, UMONS Research Institute for Health Sciences and Technology, University of Mons (UMons), 7000 Mons, Belgium; 8 Department of Otolaryngology—Head & Neck Surgery, CHU Saint-Pierre (CHU de Bruxelles), 1000 Brussels, Belgium; 9 Department of Medical and Surgical Sciences and Advanced Technologies “GF Ingrassia”, ENT Section, University of Catania, 95131 Catania, Italy; 10 Health Sciences Programme, International Center for Doctorate (EIDUDC), Universidade da Coruña (UDC), 15001 A Coruña, Spain

**Keywords:** voice prosthesis, tracheoesophageal speech, device life, laryngectomy, Provox XtraSeal, Provox Vega, head neck, cost effectiveness

## Abstract

Background: Tracheoesophageal speech with a voice prosthesis is considered the rehabilitation treatment of choice in laryngectomized patients. The main reasons for prosthesis failure are endoprosthetic leakage and periprosthetic leakage. The Provox XtraSeal^®^ stent incorporates an additional double flange on the esophageal side to prevent periprosthetic leakage. The objective of this study is to compare the duration and costs of the Provox Vega^®^ and Provox XtraSeal^®^ prostheses used in these patients in a tertiary university hospital. Materials and methods: A prospective crossover case study of laryngectomees with Provox Vega^®^ who underwent Provox XtraSeal^®^ placement due to recurrent periprosthetic leaks and decreased theoretical prosthesis life. The duration and possible factors affecting voice prostheses were studied using Kaplan–Meier curves and Cox regression. A cost-effectiveness analysis was carried out from the perspective of the Spanish National Health System with an incremental cost-effectiveness calculation. Results: A total of 38 patients were recruited, 35 men and 3 women, with a mean age of 66.26 ± 9.36 years old. Information was collected from 551 voice prostheses, 484 Provox Vega^®^ and 68 Provox XtraSeal^®^. The mean duration of Provox Vega^®^ was 119.75 ± 148.8 days and that of Provox XtraSeal^®^ was 181.99 ± 166.07 days (*p* = 0.002). The most frequent reason for replacement was endoprosthetic leakage in both groups: 283 (60.86%) in the case of Provox Vega^®^ and 29 (48.33%) in that of XtraSeal^®^ (*p* < 0.000). To obtain no cost differences (ICE ~ 0) between Provox Vega and Provox XtraSeal, the latter should cost EUR 551.63. Conclusions: The Provox XtraSeal^®^ is a cost-effective option in patients with increased prosthesis replacements due to periprosthetic leakage, reducing the number of replacements, increasing the duration of the prosthesis, and providing savings compared to Provox Vega^®^.

## 1. Introduction

Tracheoesophageal voice with voice prosthesis is the most common method of vocal rehabilitation after total laryngectomy [1]. This technique involves the placement of a one-way valve, typically a silicone or Teflon voice prosthesis (VP), between the trachea and the esophagus to allow air to enter the esophagus, which is then shaped into speech sounds by the pharyngoesophageal segment. Despite the high success rates, TEP is associated with several complications, such as VP leakage, which affects up to 30% of patients and can lead to impaired speech, social isolation, and decreased quality of life [2].

Periprosthetic leakage is one of the most common and most difficult complications to treat and is typically associated with enlargement of the tracheoesophageal puncture (TEP) that leads to the aspiration of saliva, liquid, and/or food, which flows around the voice prosthesis (VP) into the trachea and lungs. Therefore, an enlarged TEP increases the risk of pneumonia and respiratory complications due to frequent, and sometimes silent, aspiration around the VP [3]. Reduced tissue elasticity and retraction, as well as TEP enlargement itself, also increase the likelihood of spontaneous VP dislodgement and, subsequent, aspiration. While problems, such as crust formation, fungal proliferation, and endoprosthetic leakage, can be resolved simply by cleaning or replacing the prosthesis, periprosthetic leakage and TEP enlargement can be more challenging problems for physicians and can significantly reduce the quality of life of affected patients due to the high risk of aspiration and associated morbidities, loss of phonatory ability, and increased dependence on the healthcare system [4].

There is controversy about the factors that can influence the duration of the VP and the causes of periprosthetic leakage, which have been the subject of continuous study over the years [5]. In addition to individual predisposition, the influence of various factors on the duration of the VP and periprosthetic leakage has been discussed in the literature, including local inflammation or infections of the TEP, tissue atrophy around the fistula as a late effect of pre- or postoperative radiotherapy or chemoradiotherapy, the diameter or weight of the VP, the timing of the primary versus secondary TEP, the patient’s nutritional status, the duration of the follow-up, a continuous history of tobacco exposure, extensive laryngopharyngeal resection, esophageal stenosis, or the presence of diabetes, hypertension, lymph node metastasis, thyroid dysfunction, or tumor recurrence.

There appears to be a direct correlation between the incidence of enlarged TEP, subsequent periprosthetic leakage, and pharyngoesophageal reflux disease (PERD) [6]. This has been confirmed by impedance and pH measurements in several studies. Patients with PERD had a 2.3 times higher risk of TEP enlargement, with this risk being directly correlated with the severity of the reflux. Although studies have shown a decrease in the incidence of enlarged TEF and subsequent periprosthetic leakage, as well as an increase in the duration of VP in patients treated with antireflux therapy, there is still controversy regarding this issue [7,8]. However, aspects related to reflux disease that need to be considered include a higher incidence of episodes in patients undergoing laryngotracheal (LT) surgery and the possible association of reflux disease with head and neck cancer [9].

Various treatments have been proposed, including surgical closure of the TEP, which would eliminate problems associated with leakage around the prosthesis, but would also prevent voice production, negatively affecting the patient’s quality of life. Therefore, conservative methods, both surgical and non-surgical, that aim to eliminate leaks around the VP, while preserving functional voice, have gained interest over the years [10,11,12]. Although various conservative treatments have been proposed, clear guidelines for the management of this complication do not exist to date [13,14,15].

To address this issue, various modifications to VP design have been proposed, such as the Provox XtraSeal^®^ [16], which seems to reduce periprosthetic leakage and increase its duration thanks to its double esophageal flange [15]. However, the effectiveness and cost effectiveness of these newer systems compared to traditional VPs are still under investigation. Personalized medicine refers to an approach to medical care that takes into account the unique characteristics of each patient, such as their genetics, medical history, and lifestyle, to develop personalized treatment plans and care. In theory, this approach could provide more accurate and effective medical care, as it is based on specific factors of each patient rather than a general approach. It seems clear that a better understanding of the factors associated with TEP enlargement will lead to a better assessment, prevention, and treatment of this complication. Therefore, the main objective of this study is to analyze the durability of Provox XtraSeal (PVX) versus Provox Vega in patients with an increase in replacements secondary to periprosthetic leakage. Additionally, as secondary objectives, the aim is to analyze possible factors influencing the duration of VP, as well as to analyze the costs of using modified VP, such as Provox XtraSeal.

## 2. Materials and Methods

### 2.1. Setting

A cross-over prospective observational study was conducted from September 2015 to January 2023. The date range corresponds to the period of time between the placement of the first PVX prosthesis and the last one placed by the department. To minimize bias and ensure a valid control interval, the study utilized subjects as their own control [17]. The purpose of these types of studies is to determine whether there were any unusual factors that may have influenced the event being studied. Only cases were selected, and their exposures were compared with previous moments that served as a control. This approach allowed for the control of confounding factors that remained constant throughout the study and prevented biases in the selection of the controls.

### 2.2. Patients

**Figure 1:**
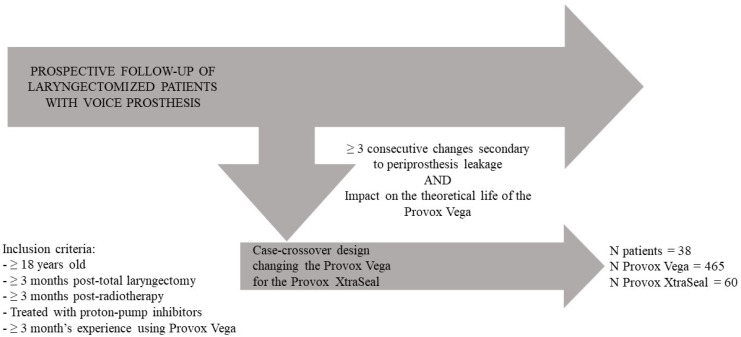
Patient selection chart.

The study recruited patients from the Otorhinolaryngology Head and Neck Surgery Department of a tertiary university hospital. The patients included were laryngectomized and were being followed up prospectively in a database that collected information on VP changes and failure causes (Figure 1) [11]. The study only included volunteer participants who were Provox Vega^®^ users and met specific criteria, including being over 18 years old, at least 3 months post-total laryngectomy, at least 3 months post-radiotherapy or chemotherapy (if applicable), at least 3 years of follow-up, treated with proton-pump inhibitors, and had at least 3 months’ experience using the Provox Vega^®^. Participants were excluded if they had medical conditions that prevented them from using the Provox system, recurrent or metastatic disease, used other phonation methods instead of VP, had functional incapacity to clean the VP independently, or had impaired cognitive ability. The study received approval from the hospital’s ethics committee (Registration code 2021/248), and informed consent was obtained from all participants.

During the study, all patients had an anterograde VP inserted, and their speech was assessed while the patients used a heat and moisture exchanger device to occlude the stoma digitally. The evaluation of the patients was conducted by an otolaryngologist and a speech therapist in all cases, who assessed the cause of the leakage and related complications. The prostheses were prescribed and used in accordance with the manufacturer’s recommendations, with PVX use criteria indicating an increase in the number of Provox Vega^®^ replacements due to periprosthetic leakage, which consecutively impacted the theoretical life of the VP, based on previous literature [11,15].

### 2.3. Statistical Analysis

The statistical analysis was conducted using Stata^®^ 14.2 for Windows (StataCorp, College Station, TX, USA). Two-tailed statistical tests were performed at a 95% confidence interval. Normality was assessed using the Kolmogorov–Smirnov test, and variances were tested using the Levene test. The quantitative variables were presented as mean ± standard deviation (SD) and median. Student’s *t*-test, Mann–Whitney, ANOVA, or the Kruskal–Wallis test were employed, as appropriate, to compare the means or medians between the groups. The qualitative variables were expressed as frequency and percentage, and the chi-square test, Fisher’s exact test, or appropriate variants were used to determine the differences between the groups. Kaplan–Meier curves and Cox proportional-hazards regression with Schoenfeld residuals were used to study the survival and factors that might impact the VP. The ongoing duration of the VP at the end of the observation period was right censored, as were the duration of the VPs that remained in situ when the patient was lost to follow-up or deceased. The event of interest was the replacement of the VP. In the univariate analyses, a two-sided significance level of 10% was used to select the variables for inclusion in the multivariate models. The independent variables included age, sex, type of puncture, pT stage, pN stage, tumor location, tumor stage, and neck dissection, or complementary treatment with radiotherapy.

### 2.4. Costs Analysis

The cost-effectiveness analysis (CEA) included the direct medical costs, such as the cost of the prostheses, which were obtained from the hospital’s economic department (Spanish Public National Health System, 2023). The cost of each Provox Vega^®^ was EUR 363 and, for Provox XtraSeal, a range between EUR 400 and EUR 600 was selected depending on the health center assessed. The surgical procedures, and follow-up visits were excluded in order to extrapolate the results to other settings. This is because each center uses its own care protocol, and there is currently no consensus on this subject [13,14].

The expected annual number of VP replacements was estimated using the information obtained from the study sample. The incremental cost-effectiveness ratio (ICE) was calculated by dividing the difference in costs between the PVX and Provox Vega^®^ by the difference in the expected number of annual VP replacements between the two devices. The ICE represents the additional cost required to achieve one additional replacement of the VP with the PVX compared to the Provox Vega^®^. The expected duration for Provox Vega^®^, according to the existing literature [11,15], was 3.5 changes per year. This expected number of changes is based on our previous database with an equivalent sample population and should, therefore, accurately reflect the reality of the duration of both VPs.

## 3. Results

### 3.1. Descriptive Analysis

A total of 38 patients were recruited, 35 men and 3 women, with a mean age of 66.26 ± 9.36 years old. Information was collected on 551 voice prostheses, 483 Provox Vega^®^ and 68 Provox XtraSeal^®^. The remaining collected variables are described in Table 1, with the reference unit being the prosthesis. Significant differences were found in the multivariate analysis when comparing Provox Vega to PVX regarding radiation therapy (*p* = 0.006), right neck dissection (*p* = 0.000), and the laterality of the cervical dissection (*p* = 0.012).

### 3.2. Replacement Reasons

A total of 525 prosthesis replacements were evaluated, with 465 (88.57%) Provox Vega and 60 (11.43%) Provox XtraSeal. The most common reason for replacement in both types of voice prosthesis was endoprosthesis leakage (*n* = 312; 59.43%), with 283 (60.86%) instances occurring with Provox Vega and 29 (48.33%) with Provox XtraSeal. All reasons for replacement were documented. The second most frequent reason for replacement was periprosthetic leakage in Provox Vega (*n* = 97; 20.86%), compared to extrusion in Provox XtraSeal (*n* = 17; 28.33%). Significant differences were found in the distribution of the replacement reasons, according to the type of voice prosthesis (*p* = 0.000). The remaining reasons for voice prosthesis replacement are summarized in Table 2.

### 3.3. Duration and Multivariate Cox Proportional Hazards Regression Analysis

**Figure 2:**
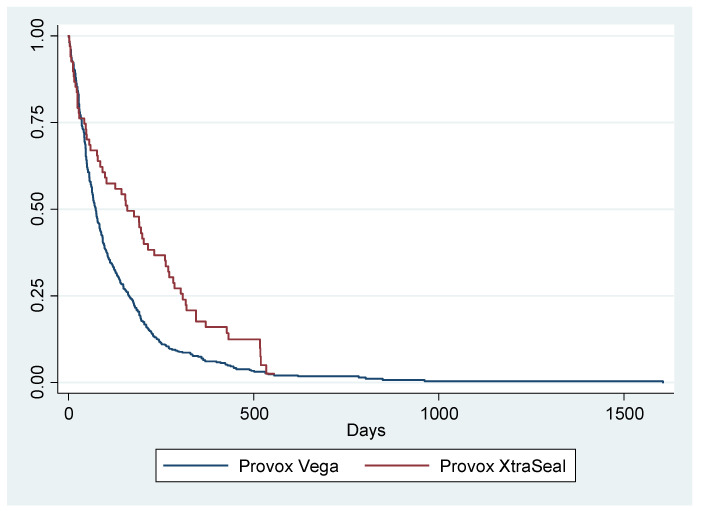
Prosthetic duration curves: Provox Vega versus Provox XtraSeal (*p* = 0.002).

The mean duration of the Provox Vega^®^ was 119.75 ± 148.8 days (median: 73 days), while that of Provox XtraSeal^®^ was 181.99 ± 166.07 days (median: 159 days) (*p* = 0.002). The prosthetic duration curves can be seen in Figure 2.

After analyzing possible interactions between the variables, confounding factors, and performing the likelihood-ratio test, the resulting multivariate model included the type of prosthesis, gender, age, tumor location, tumor stage, and type of cervical dissection performed. The multivariate Cox analysis (Table 3) showed that Provox XtraSeal was a protective factor against replacement (*p* = 0.003), as well as the female gender (*p* = 0.000), cervical dissection compared to no dissection (unilateral or bilateral, *p* = 0.000), age (*p* = 0.013), and tumor stage. The supraglottic location appeared to be a contributing factor to prosthesis replacement (*p* = 0.005).

### 3.4. Cost-Effectiveness Analysis

The annual replacement rates for the VP were 3.05 for Provox Vega, and 2.01 for Provox XtraSeal. In the case of Provox Vega, this rate represents a 12.8% decrease in the number of predicted replacements based on previous literature [11]. In the case of Provox XtraSeal, it represents a decrease of 42.6%. To obtain no cost differences (ICE ~ 0) between Provox Vega and Provox XtraSeal, the latter should cost EUR 551.63 (Table 4).

**Figure 3:**
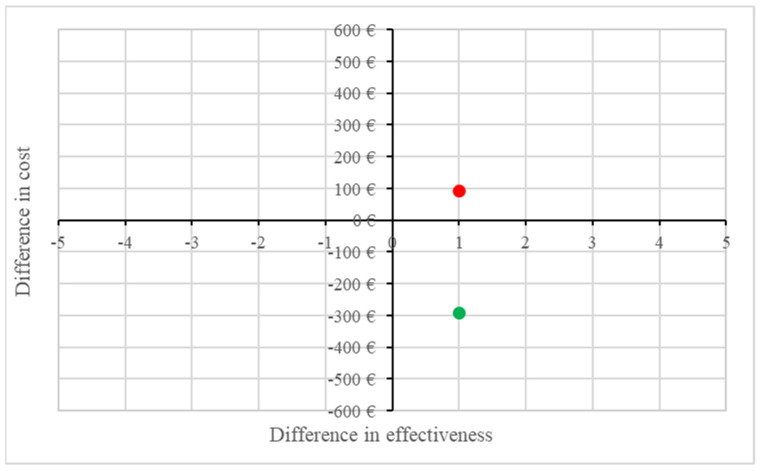
Cost-effectiveness plane. Note the green dot position of the Provox XtraSeal^®^ intervention in area II (more effective and less expensive interventions) versus the red dot position in area I (more expensive but equally as effective).

Considering the proposed price range for Provox XtraSeal, between EUR 400 and EUR 600, the incremental cost-effectiveness ratio (ICE) between the two voice prostheses was EUR −291.80 for the lower cost scenario, and EUR 93.07 for the higher cost scenario (Figure 3).

## 4. Discussion

In 2016, the Provox^®^ Vega™ XtraSeal™ appeared on the market to treat periprosthetic leaks and TEP enlargement, incorporating a double flange on the esophageal side of the VP in order to prevent such leaks and maintain the patient’s ability to communicate. This is simply a modification of the Provox Vega in which this second flange is angled, thin and flexible, improving its adherence to the surface around the tracheoesophageal puncture tract. Despite the increasingly widespread use of this new type of VP, there are few studies that support its usefulness in the prevention or treatment of periprosthetic leaks [15]. The aim of this study was to evaluate the usefulness of PVX in patients with periprosthetic leaks, as well as to represent the first cost-utility study of this type of device.

Regarding the mean duration of the VP, ranges between 66 and 124 days [11,18,19] have been reported for Provox Vega^®^, and 114 and 176 days for Provox XtraSeal^®^ [11,15]. These results corroborate those found in the present study, suggesting that the Provox XtraSeal^®^ prosthesis is useful in preventing periprosthetic leakage. Regarding the reasons for replacement, endoprosthetic leakage remains the most frequent cause in both types of voice prosthesis. Periprosthetic leakage is the second most common reason for replacement in Provox Vega, which is consistent with previous literature [11,18,20,21]. In the case of PVX, the second most frequent cause of prosthetic replacement was found to be extrusion, which is reported in the literature with a range of 3% to 11% [2], relegating periprosthetic leakage to third position. These findings point to a notable efficacy in reducing periprosthetic leakage by PVX prosthesis, as well as the correct selection of patients. It has been suggested that this higher rate of extrusion replacements for PVX may be due to a possible relationship with the greater technical difficulty when placing [15].

Cost-effectiveness analysis supports the use of PVX as a method to reduce periprosthetic leaks, as long as the cost of the PVX remains below EUR 551.63. At a higher cost for the PVX, its effectiveness in reducing periprosthetic leaks is maintained, but the cost compared to Provox Vega increases. This aspect has been a topic of debate in the field of clinical management over the years. Cost-effectiveness analysis provides valuable information for decision-making, as it considers both the costs and effectiveness of interventions. However, it is important to consider other factors as well, such as patient preferences, clinical outcomes, and the overall impact on quality of life, when making decisions about the use of PVX or any other intervention. To evaluate the true benefit of PVX, despite it potentially being a more expensive intervention, it is necessary to consider the morbidity and costs associated with an increase in periprosthetic leaks in a personalized and individualized approach for each patient. Assessing these factors is crucial in determining the overall value and cost effectiveness of PVX compared to other options.

Many factors that may influence the rate of prosthesis replacement have been proposed in different publications; some of them are: radiotherapy treatment, the extent of the surgery, the time of the tracheoesophageal puncture, the experience of the team, the presence of a speech therapist expert, age, gastric reflux, nutritional status, or the extent of nodal involvement [5,6,8,22]. In this regard, our results are in agreement with previous publications. Despite the lack of statistical significance, age seems to play a protective role against replacements. One theory is that older age corresponds to less frequent use of the voice prosthesis, resulting in less deterioration. On the other hand, supraglottic extension, which involves greater mucosal resection during total laryngectomy, appears to correlate with a higher number of prosthesis replacements. The other factors found to be protective in the multivariate analysis (tumor stage, cervical dissection) should be interpreted with caution. It seems plausible that the lower the tumor stage, the less resection is required, and the more pharyngeal mucosa remains, thus representing a protective factor. This should be similar in the case of the pT stage, but in our sample the opposite is true. Lower stages (pT2) seem statistically to significantly increase the number of replacements. It is important to note that the study population represents a subgroup with an increased number of replacements due to periprosthetic leaks and does not constitute a representative sample of the total laryngectomy patient population with voice prostheses. More studies are necessary in this line of research to elucidate, with greater precision, the causal relationship and the effect of these different factors [5,15].

In this study, it was not possible to evaluate radiotherapy as a predisposing factor for an increased number of replacements or complications at the TEP level, due to the differences found between the groups (Table 1). Larger population studies are needed to draw inferences in this regard. Continuing in this line, an aspect that has received limited attention in the literature and requires further investigation is the potential influence of repeated voice prosthesis replacements on TEP and its enlargement. As mentioned earlier, radiated tissue is atrophic and poorly vascularized. Patients who require a higher number of replacements than expected may experience microtrauma due to the insertion of a new voice prosthesis. This unavoidable microtrauma, given the current method of insertion, could contribute to the development of an enlarged TEP. Further research is needed to better understand this relationship and its implications for patient care.

Among the strengths of this study is the extensive collection of voice prostheses through the prospective follow-up, enabling the documentation of all the reasons for replacement (Table 2). Additionally, this article represents the largest sample of Provox XtraSeal studied to date. The meticulous selection of patients, who were candidates for using PVX due to recurrent periprosthetic leaks, is also noteworthy. We consider the management by an expert team and the appropriate selection of candidates based on their comorbidities to be important factors contributing to the study’s strength [13,14].

The present study has some limitations, such as its observational design and the low number of patients included in the PVX group, which may be attributed to the strict patient selection criteria. The data may not be easily generalized to other populations due to the lack of representativeness, differences in management protocols across hospitals or countries, and variations in the experience of the medical teams. Another limitation to the present study stems from the use of cost-effectiveness analysis, which is often criticized for its subjectivity in defining the measures of effectiveness. It might be worth considering cost-utility research to obtain results using a widely known and utilized unit of effect, such as quality of life. However, this would require the implementation of specific and validated questionnaires for this particular patient population, which are not routinely used in clinical practice or research [23,24,25].

## 5. Conclusions

This study represents an important contribution to the literature, as it is the first prospective case-crossover study comparing Provox Vega^®^ and Provox XtraSeal^®^ voice prostheses. The long-term follow-up, multivariate regression analysis, and cost-effectiveness analysis performed in this study provide robust evidence.

The results of the study strongly suggest that the Provox XtraSeal^®^ prosthesis is effective in preventing periprosthetic leakage compared to Provox Vega^®^. This finding supports the use of Provox XtraSeal^®^ in patients who require frequent prosthesis changes due to periprosthetic leakage. By reducing the number of changes needed, the Provox XtraSeal^®^ prosthesis offers a cost-effective alternative.

The positive cost-effectiveness relationship of the Provox XtraSeal^®^ prosthesis implies that the benefits gained from using this prosthesis outweigh the associated costs. This is particularly relevant for patients who experience recurrent periprosthetic leakage, as the Provox XtraSeal^®^ prosthesis can help minimize the need for frequent replacements and the associated costs.

Overall, the findings in this study provide valuable evidence supporting the efficacy and cost effectiveness of the Provox XtraSeal^®^ prosthesis as a suitable option for patients requiring frequent changes due to periprosthetic leakage.

## Figures and Tables

**Table 1:** Descriptive analysis, taking the prosthesis as a reference.

	Provox Vega	%	Provox XtraSeal	%	Total	%	*p*-Value
Radiotherapy							
Yes	215	46.24	39	65.00	254	48.38	0.006
No	250	53.76	21	35.00	271	51.62	
TEP							
Primary	274	58.92	41	68.33	315	60.00	0.161
Secondary	191	41.08	19	31.67	210	40.00	
Neck dissection							
No	85	18.28	9	15.00	94	17.90	0.012
Unilateral	69	14.84	18	30.00	87	16.57	
Bilateral	311	66.88	33	55.00	344	65.52	
Left neck dissection							
No	120	25.81	21	35.00	141	26.86	0.117
Functional	323	69.46	34	56.67	357	68.00	
Radical	22	4.73	5	8.33	27	5.14	
Right neck dissection							
No	119	25.59	15	24.19	134	25.43	0.000
Functional	337	72.47	38	61.29	375	71.16	
Radical	9	1.94	9	14.52	18	3.42	
Tumor location							
Glottic	103	22.15	17	28.33	126	22.83	0.624
Supraglottic	157	33.76	16	26.67	179	32.43	
Transglottic	126	27.10	16	26.67	149	26.99	
Hypopharynx	79	16.99	11	18.33	98	17.75	
pT stage							
T1	33	7.10	5	8.33	40	7.25	0.859
T2	61	13.12	10	16.67	76	13.77	
T3	240	51.61	29	48.33	278	50.36	
T4a	131	28.17	16	26.67	158	28.62	
pN stage							
N0	302	64.95	40	66.67	357	64.67	0.576
N1	52	11.18	3	5.00	58	10.51	
N2a	15	3.23	3	5.00	21	3.80	
N2b	62	13.33	9	15.00	74	13.41	
N2c	6	1.29	0	0.00	7	1.27	
N3b	28	6.02	5	8.33	35	6.34	
Tumor stage							
I	24	5.16	1	1.67	26	4.71	0.152
II	23	4.95	3	5.00	27	4.89	
III	159	34.19	23	38.33	187	33.88	
IV A	204	43.87	20	33.33	237	42.93	
IV B	55	11.83	13	21.67	75	13.59	

**Table 2:** Voice prosthesis replacement reasons (*p* = 0.000).

	Provox Vega	%	Provox XtraSeal	%	Total	%
Endoprosthetic	283	60.86	29	48.33	312	59.43
Periprosthetic	97	20.86	7	11.67	104	19.81
Fungic colonization	10	2.15	1	1.67	11	2.10
Extrusion	46	9.89	17	28.33	63	12.00
Peri + Endoprosthetic	16	3.44	1	1.67	17	3.24
Deterioration	0	0.00	2	3.33	2	0.38
No phonation	13	2.80	3	5.00	16	3.05
Total	**465**	**88.57**	**60**	**11.43**	**525**	

**Table 3:** Multivariate Cox regression model.

	Hazard Ratio	Standard Error	*p*-Value	95%CI	
XtraSeal	0.66	0.09	**0.003**	0.5	0.87
Secondary TEP	0.9	0.09	0.297	0.98	1
Neck dissection					
Unilateral	0.5	0.1	**0.000**	0.33	0.73
Bilateral	0.51	0.08	**0.000**	0.37	0.69
Tumor stage					
II	0.13	0.08	**0.000**	0.04	0.43
III	0.5	0.24	0.155	0.2	1.3
IVA	0.53	0.23	0.149	0.22	1.26
IVB	0.37	0.2	0.067	0.12	1.07
Location					
Supraglottic	1.38	0.21	**0.03**	1.03	1.86
Transglottic	1.06	0.17	0.684	0.79	1.45
Hypopharynx	0.95	0.22	0.837	0.6	1.51
pT stage					
T2	2.21	0.82	**0.033**	1.07	4.58
T3	1.82	0.66	0.096	0.9	3.68
T4a	0.95	0.22	0.486	0.61	2.78
Age	0.99	0.01	0.104	0.97	0.99

**Table 4:** Cost-effectiveness analysis targeting equal cost between Provox Vega and XtraSeal.

		Provox Vega	Provox XtraSeal
Changes per year	Predicted [11,18]	3.50	3.50
	Outcome	3.05	2.05
Effectiveness		0.45	1.49
		**1.04**	
Price (EUR)	Predicted [11,18]	1269.08	1928.55
	Outcome	EUR 1107.15	1107.14
	Difference	−161.93	−821.41
		**−938.86**	
Mean cost-effectiveness (MCE)		EUR 2481.92	EUR 743.52
**Incremental cost-effectiveness (ICE)**		**EUR −0.01**	

## Data Availability

Data available on request due to restrictions, e.g., privacy or ethical.
